# Bio-Inspired Presentation Attack Detection for Face Biometrics

**DOI:** 10.3389/fncom.2019.00034

**Published:** 2019-05-28

**Authors:** Aristeidis Tsitiridis, Cristina Conde, Beatriz Gomez Ayllon, Enrique Cabello

**Affiliations:** Computer Science and Statistics, King Juan Carlos University, Móstoles, Spain

**Keywords:** face biometrics, presentation attack detection, anti-spoofing, multiple sensor fusion, biologically-inspired biometrics

## Abstract

Today, face biometric systems are becoming widely accepted as a standard method for identity authentication in many security settings. For example, their deployment in automated border control gates plays a crucial role in accurate document authentication and reduced traveler flow rates in congested border zones. The proliferation of such systems is further spurred by the advent of portable devices. On the one hand, modern smartphone and tablet cameras have in-built user authentication applications while on the other hand, their displays are being consistently exploited for face spoofing. Similar to biometric systems of other physiological biometric identifiers, face biometric systems have their own unique set of potential vulnerabilities. In this work, these vulnerabilities (presentation attacks) are being explored via a biologically-inspired presentation attack detection model which is termed “BIOPAD.” Our model employs Gabor features in a feedforward hierarchical structure of layers that progressively process and train from visual information of people's faces, along with their presentation attacks, in the visible and near-infrared spectral regions. BIOPAD's performance is directly compared with other popular biologically-inspired layered models such as the “Hierarchical Model And X” (HMAX) that applies similar handcrafted features, and Convolutional Neural Networks (CNN) that discover low-level features through stochastic descent training. BIOPAD shows superior performance to both HMAX and CNN in all of the three presentation attack databases examined and these results were consistent in two different classifiers (Support Vector Machine and *k*-nearest neighbor). In certain cases, our findings have shown that BIOPAD can produce authentication rates with 99% accuracy. Finally, we further introduce a new presentation attack database with visible and near-infrared information for direct comparisons. Overall, BIOPAD's operation, which is to fuse information from different spectral bands at both feature and score levels for the purpose of face presentation attack detection, has never been attempted before with a biologically-inspired algorithm. Obtained detection rates are promising and confirm that near-infrared visual information significantly assists in overcoming presentation attacks.

## Introduction

Biometrics have a long history of existence and usage in various security environments. Modern biometric systems utilize a variety of physiological characteristics also known as “biological identifiers.” For example, non-intrusive biometric patterns extracted from a finger, palm, iris, voice, gait (and their fusion in multimodal biometric systems), can provide a wealth of identity information about a person. Face biometrics in particular, pose a challenging practical problem in computer vision due to dynamic changes in their settings such as fluctuations in illumination, pose, facial expressions, aging, clothing accessories, and other facial feature changes such as tattoos, scars, wrinkles and piercings. The main advantage of face biometric applications is that they can be deployed in diverse environments at low cost (in many cases, a simple RGB camera is sufficient) without necessitating substantial participation and inconvenience from the public. Public acceptance of face biometrics is also the highest amongst all other biological identifiers. Modern day applications making extensive use of face biometric systems include, mobile phone authentication, border or customs control, visual surveillance, police work, and human-computer interaction. Regardless of the numerous practical challenges in this field, face biometrics still remain a heavily researched topic in security systems.

Face biometric systems are susceptible to intentional changes in facial appearance or falsification of photos in official documents known as, “presentation attacks.” For example, impostors may acquire a high quality face image of an individual and manipulate it either printed on paper, on a mask or even on a smartphone display to deceive security camera checkpoints. The significant reduction in high-definition portable camera size also means that impostors have easy access to tiny digital cameras that discretely or secretively capture face images of unsuspecting individuals. Moreover, with the vast online availability of face images in public or social media, it is relatively easy to acquire and reproduce a person's image without their consent. “Presentation Attack Detection (PAD)” or less formally known “anti-spoofing,” engulfs the detection of all spoofing attempts made on biometric systems. Therefore, accurate and fast PAD is an important problem for authentication systems across many platforms and applications (Galbally et al., 2015) in the fight against malicious security system attacks. Basic face presentation attacks often are: (a) printed face on a paper sheet. Sometimes a printed face is shown with eyes cropped out so that the impostor's eyes blink underneath. (b) Digital face displayed on a screen from digital devices such as tablets, smartphones, and laptops. This kind of face presentation attacks can be static or video. In video attacks facial movements, eye blinking, mouth/lip movements or expressions are usually simulated through a short video sequence. (c) A 3D mask (paper, silicon, cast, rubber etc.) specifically molded for a targeted face. In addition, impostors may also try identity spoofing by using more sophisticated appearance alteration techniques or their combinations: (1) Glasses corrective or otherwise and/or contact lenses with possible color change. (2) Hairstyle, change in color, cut/trim, hair extensions etc. (3) Make-up or fake facial scars. (4) Real and/or fake facial hair. (5) Facial prosthetics and/or plastic surgery.

Presentation attacks in images can be detected from anomalies in image characteristics such as liveness, reflectance, texture, quality, and spectral information. Sensor-based approaches are considered efficient strategies to investigate such image characteristics and naturally involve the usage (and fusion) of various camera sensors that capture minute discrepancies. A sensor-based method that uses a light field camera sensor with 26 different focus measures together with image descriptors (Raghavendra et al., 2015) reported promising PAD scores. With the aid of infrared sensors authors in Prokoski and Riedel (2002) analyzed facial thermograms for rapid, and varied illumination environments. Similar thermography methods were presented in Hermosilla et al. (2012) and Seal et al. (2013). Motion-based techniques are mostly employed in video sequences to detect motion anomalies between frames. Some representative methods of this type of PAD algorithms used Eulerian Video Motion Magnification (Wu et al., 2012), Optical Flow (Anjos et al., 2014), and non-rigid motion with face-background fusion analysis (Yan et al., 2012). Liveness-based approaches extract image features that focus on the liveness phenomena of a particular subject. Using this approach, algorithms scan liveness patterns in certain facial parts such as facial expressions, mouth or head movements, eye blinking, and facial vein maps (Pan et al., 2008; Chakraborty and Das, 2014). Texture based methods investigate texture, structure and overall shape information of faces. In conventional terms, commonly used texture-based methods rely on Local Binary Patterns (Maatta et al., 2011; Chingovska et al., 2012; Kose et al., 2015), Difference of Gaussians (Zhang et al., 2012) and Fourier frequency analysis (Li et al., 2004). For quality characteristics, a notable image quality method in Galbally et al. (2014) proposed 25 different image quality metrics as extracted between real and fake images in order to train classifiers which are then used for the detection of potential attacks.

In today's society, face perception is extremely important. In the distant past, our very survival in the wild depended on our ability to collaborate collectively as species. As a consequence, the human brain over the millennia has evolved to perform facial perception in an effortless, rapid and efficient manner (Ramon et al., 2011). The ever increasing requirements in complexity, power and processing speed, have motivated the biometric research community to explore new ways of optimizing facial biometric systems. Therefore, it should not come as a surprise that biology has recently become a valuable source of inspiration for fast, power efficient and alternative methods (Meyers and Wolf, 2008; Wang et al., 2013).

The fundamental biologically-motivated vision architecture consists of alternating hierarchical layers mimicking the early processing stages of the primary visual cortex (Hubel and Wiesel, 1967). It is established from past research that as visual stimuli are transmitted up the cortical layers (from V1–V4), visual information progressively exhibits a combination of selectivity and invariance to object translations such as size, position, rotation, depth etc. In the past, there have been many vision models and variants inspired from this approach such as the “Neocognitron” (Fukushima et al., 1980), “Convolutional neural network” (LeCun et al., 1998), and “Hierarchical model and X” (Riesenhuber and Poggio, 2000). Over the years, these models have performed incredibly well in many object perception tasks and today are recognized as equal alternatives to statistical techniques. In face perception, biologically-inspired methodologies have been applied successfully for some years and have proven reliable as well as accurate (Lyons et al., 1998; Wang and Chua, 2005; Perlibakas, 2006; Rose, 2006; Meyers and Wolf, 2008; Pisharady and Martin, 2012; Li et al., 2013; Slavkovic et al., 2013; Wang et al., 2013).

There are many common characteristics in biologically-motivated algorithms and perhaps the most important aspect is the extensive use of texture-based features in either 2D or 3D images. Reasons for designing a biologically-inspired model would be its projected efficiency, parallelization and speed in extremely demanding biometric situations. Contemporary state-of-the-art methods are efficient in selected environments with high availability of data but sifting each frame with laborious and lengthy CNN training, sliding windows or pixel-by-pixel approaches requires an incredible amount of available resources such as storage capacity, processing speed and power. Nevertheless, biologically-inspired systems have almost entirely been expressed by deep learning CNN architectures. In Lakshminarayana et al. (2017), spatio-temporal mappings of faces extraction is followed by a CNN schema, and discriminative features for liveness detection were subsequently acquired. This approach produced impressive results on the databases examined but their setup relied solely on video sequences which penalize processing speed and are not always available in the real world, especially in border control areas where a single image should suffice. Other CNN models (Alotaibi and Mahmood, 2017; Atoum et al., 2017; Wang et al., 2017) explored depth perception prior to application of a CNN that distinguished original vs. impostor access attempts. In Alotaibi and Mahmood (2017), depth information was produced with a non-linear diffusion method based on an additive operator splitting scheme. Even though only a single image was required in this work, the use of only one database (and the high error rates in the Replay-Attack database) did not entirely reveal the potential of this approach. Another CNN approach was presented in Atoum et al. (2017) where a two-stream CNN setup for face anti-spoofing was employed by extracting local image features and holistic depth maps from face frames of video sequences. Experimentation with this CNN setup showed reliable results with a significant cost on practicality i.e., training two separate CNNs along with all intermediate processing steps. In Wang et al. (2017), a representation joining together 2D textual information and depth information for face anti-spoofing was presented. Texture features were learned from facial image regions using a CNN and face depth representation was extracted from Kinect images. The high error rates and limited experimentation procedure made their findings rather questionable. Finally, in Liu et al. (2018) a CNN-RNN (Recursive Neural Network) model was used to acquire face depth information with pixel-wise supervision, by estimating remote photoplethysmography signals together with sequence-wise supervision. The accuracy of this method relied heavily on the number of frames per video which makes this approach computationally heavy.

Overall, Convolutional Neural Network approaches and the manner in which they are executed or accelerated in hardware is a big subject of debate in our world today. They require large amounts of resources in hardware, software and energy to be effectively trained. However, since end-users have different hardware/software configurations, no particular effort was given to hardware optimization or software acceleration. The investigation of a biologically-inspired PAD secure system was developed as part of two funded projects, the European project ABC4EU and the Spanish national project BIOINPAD. End-users in both projects (i.e., the Spanish national police, Estonian police, Rumanian Border Guard) were interested in a new approach to the PAD problem.

Over the years, bio-inspired systems have received significant interest from the computer vision community because their solutions can relate to real-world human experiences. Thus, the main research contribution of this work has been the introduction of a system that handles video presentation attack detection from a biologically-inspired perspective. A system that has a straightforward and simple architecture able to cope with visual information from a single frame at high precision rates. Our design focus has been the development of a bio-inspired system with a clear structure and relatively little effort. In addition, this paper summarizes precision rate results obtained during our research and compares them against other known models to enhance the comparative scope and understanding. The system has been evaluated with different databases in the visible, and near-infrared (and their fusion) spectral regions. This is illustrated over several sections of this article which is organized in the following way. In section Methodology and BIOPAD's structure, definitions and methodology that have led us to the development of the BIOPAD model are discussed, followed by a detailed explanation of the model's structure. Furthermore, in that section, we demonstrate the biologically-inspired techniques used, the model's general layout, and individual layer functionality. Section Experiments describes all databases used (section Databases), explains our biometric evaluation procedures (section Presentation attack results) and analyses all experiments conducted for the BIOPAD, Hierarchical Model And X (HMAX) and CNN (AlexNet) models. Section Experiments is further divided into visible (section Visible spectrum experiments) and near-infrared (section Near-infrared experiments and cross-spectral fusion) experiments for a better comparison between the two approaches explored. Finally, the last section summarizes all of our conclusions in this research work.

## Methodology and BIOPAD's Structure

In the first part of this section, the overall layered structure is described, followed by the biologically-inspired concepts that have been used as core mechanisms in BIOPAD. In the last section, each layer is individually explored, along a full explanation of its operation in a pseudo-like manner.

### Center-Surround and Infrared Channels

Mammals perceive incoming photons through the retina in their eyes. The number of individual photoreceptors in the retina of the human eye varies from person to person and in the same person from time to time, but on average each eye consists of ~5 million cones, 120 million rods and 100 thousand photosensitive retinal ganglion cells (Goldstein, 2010).

**Figure 1:**
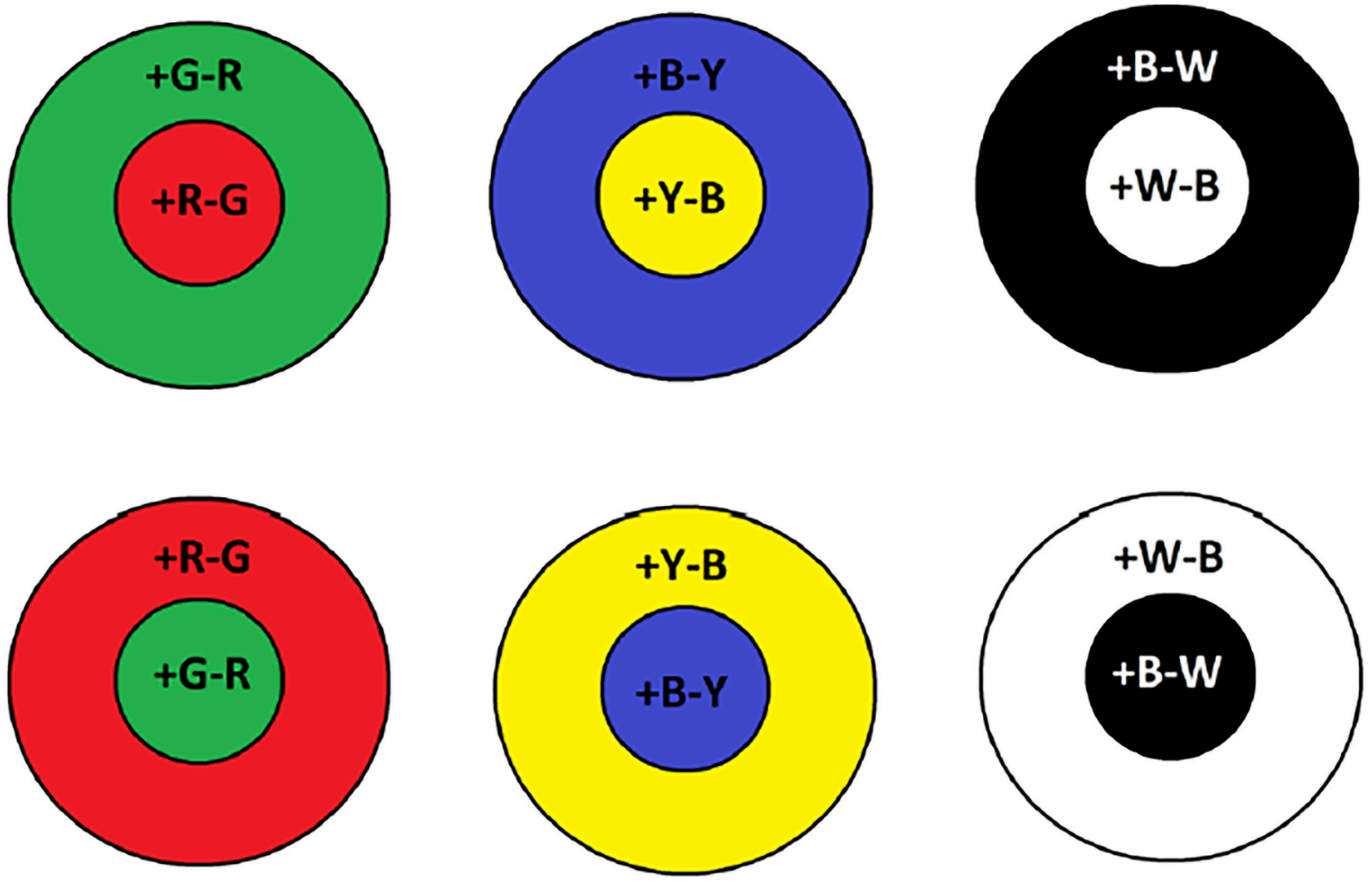
Examples of on-center and off-center receptive fields for color opponency channels. Plus sign indicates whether the particular color is on and the minus off.

In the human retinae, rod photoreceptors peak at ~500 nm, they are slow response receptors, come in small numbers, possess large receptive fields, and are suitable for dark environments i.e., night time. However, cone receptive fields are narrower and are tuned to different wavelengths of light. They are considerably greater in numbers than rods and hence, are responsible for visual acuity. Bipolar retinal cells bear the task of unifying incoming visual information from cones and rods (Engel et al., 1997). Furthermore, on-center and off-center bipolar cells operate in a center-surround process between red-green and blue-yellow wavelengths. For example, on-center Green-Red (RG) bipolar cells are going to maximally respond when red hits the center of their receptive field only and are inhibited when green is at their surrounding region. Vice versa, this operation is reversed for an off-center RG bipolar cell where excitation only occurs when the detectable green wavelength is incident in the surrounding region. As shown in Figure 1, this can be further applied for the blue-yellow and lightness channels. The color opponent space is defined by the following equations (Van De Sande et al., 2010):

(1) O1=(R−G)/√2

(2) O2=(R+G−2B)/√6

(3) O3=(R+G+B)/√3

The O3 opponent channel is the intensity channel and color information is conveyed by channels O1 and O2. In BIOPAD, when the input image is in RGB, all three opponent channels are processed simultaneously and in order to make use of the available infrared information, an additional channel NIR is added in the fourth channel dimension.

The use of infrared or thermal imaging alongside the visible spectrum, has been the subject of investigation many times in the past (Kong et al., 2005) and Gabor filters with near-infrared data have been applied together with computer vision algorithms (Prokoski and Riedel, 2002; Singh et al., 2009; Zhang et al., 2010; Chen and Ross, 2013; Shoja Ghiass et al., 2014). However, the use of infrared spectra in presentation attack detection using a biologically-motivated model, to our knowledge, is a first with this research work.

**Figure 2:**
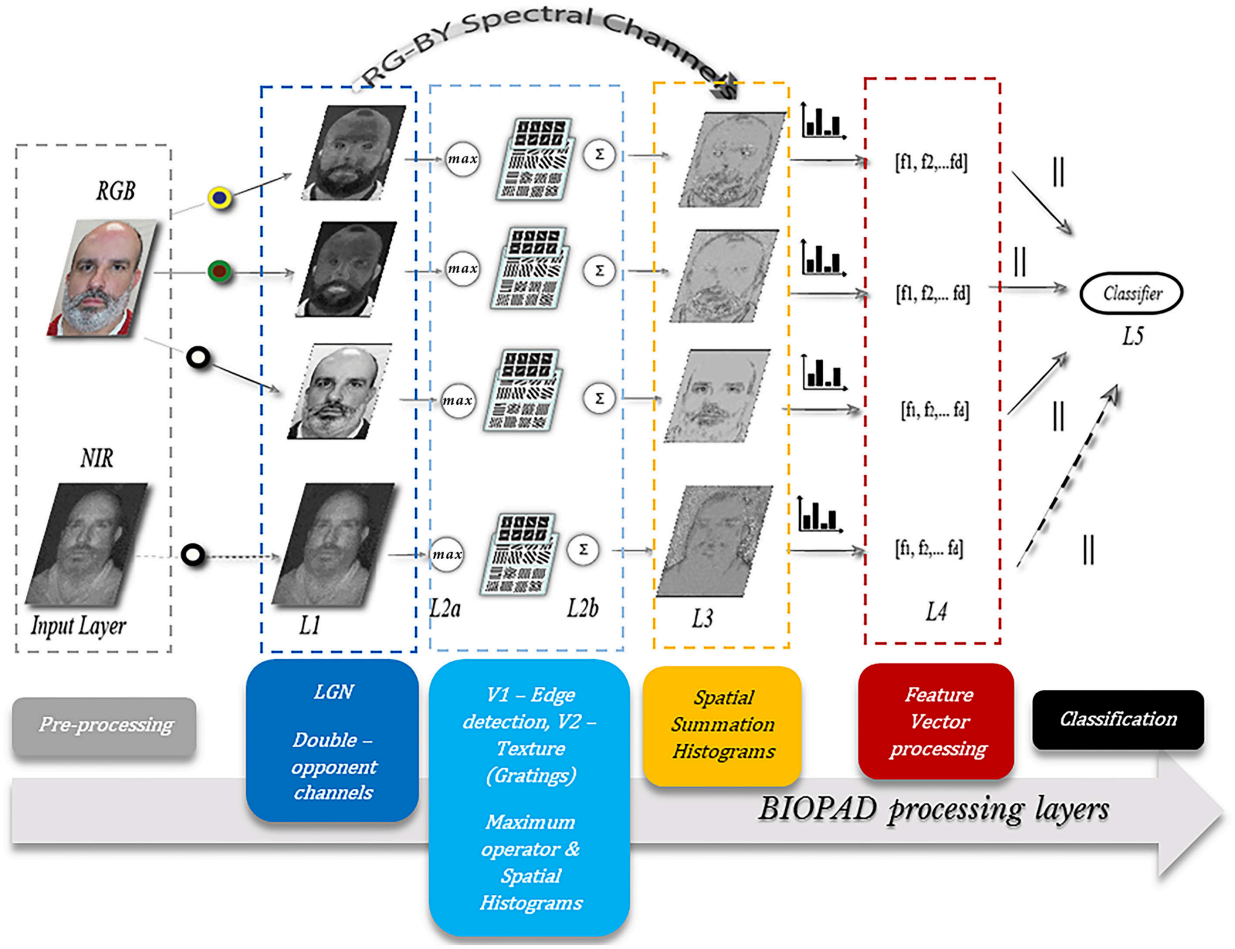
The proposed model structure. Several layers L1 to L5 progressively process spatial and spectral facial features. All participants gave written informed consent for the publication of this manuscript.

The actual infrared range of wavelengths can be huge, spanning from 7 microns all the way up to 300 microns and generally these bands, are undetectable to the human eye. However, there is evidence that infrared wavelengths up to 10 microns under certain circumstances are detectable by humans as visible light (Palczewska et al., 2014). From a biological perspective, the exact mechanism of near-infrared perception in the visual cortex is unknown. In BIOPAD and at low feature level, it is treated as an additional channel input from the retina, with a range of normalized pixel values as provided by the sensor (Figure 2). Infrared data acquisition and sensor information is shown in section Presentation attack results.

### Area V1—Edge Detection

As visual signals travel to the primary visual cortex through the lateral geniculate nucleus, area V1 orientation selective simple cells process incoming information (Hubel and Wiesel, 1967) from the retinae and perform basic edge detection operations for all subsequent visual tasks. They serve as the building block units of biological vision. It is already well-established from literature that orientation selectivity in V1 simple cells can be precisely matched by Gabor filters (Marcelja, 1980; Daugman, 1985; Webster and De Valois, 1985).

A Gabor filter is a linear filter which is defined as the product of a sinusoid with a 2D Gaussian envelope and for values in pixel coordinates *(x, y)*, it is expressed as:

(4) $$\text{G}\left(\text{x},\text{ y}\right)=\exp\left(-\frac{{\text{X}}^{2}\text{+}\gamma^{\text{2}}{\text{Y}}^{2}}{\text{2}\sigma^{\text{2}}}\right)\text{ cos }\left(\frac{\text{2}\pi }{\lambda }\right)\text{ X}$$

(5) X=x cos θ−y sin θ

(6) Y=−x sin θ+y cos θ

In Equation 5, γ is the aspect ratio and in this work is set to 0.3. Parameter λ is known as the wavelength of the cosine factor and together with the effective width, parameter σ, specify the spatial tuning accuracy of the Gabor filter. Ideally, to optimize the extraction of contour features from V1 units for a particular set of objects, some form of learning is necessary to isolate an optimum range of filters. However, this process adds complexity and it is time-consuming since it requires a huge number of samples, as experiments on convolutional neural networks have shown in literature. In order to avoid this step, Gabor filter parameters are hardcoded directly into our model following parameterization sets that have been identified from past studies. Two different parameterization settings have been considered (Serre and Riesenhuber, 2004; Lei et al., 2007; Serrano et al., 2011). Our preliminary experiments have shown that the two particular Gabor filter parameterization ranges, have no noticeable effect on PAD results. Thus, we chose the parameterization values given (Serrano et al., 2011).

Additionally, it is known that V1 cell receptive field sizes vary considerably (McAdams and Reid, 2005; Rust et al., 2005; Serre et al., 2007) to provide a range of thin to coarse spatial frequencies. Similarly, four different receptive field sizes were used here with pixel dimensions 3 × 3, 5 × 5, 7 × 7, and 9 × 9. Coarser features are handled by area V2, explained in the next section.

### Area V2—Texture Features

In general, the significance of textural information is sometimes neglected or even downplayed in past biologically-inspired vision models. In face biometrics, as explained previously in the introductory section, there is a long list of texture-based presentation attack detection models and texture information is considered a crucial feature against attacks.

The role of cortical area V2 in basic shape and texture perception is essential. V2 cells share many of the edge properties found in V1. Nevertheless, V2 cell selectivity has broader receptive fields and is attuned to more complex features compared with V1 cells (Hegdé and Van Essen, 2000; Schmid et al., 2014). In addition to broader spatial features, this layer processes textural information and is therefore capable of expressing the different nature of surfaces. This is a crucial advantage in face presentation attack detection where there is a wealth of information hidden within the texture of faces, facial features or face attacks. For example, texture of beards, skin, and glasses can prove a valuable feature against spoofing attacks mimicking their nature.

V2 cells are effectively expressed by a sinusoidal grating cell operator though other shape characteristics also correspond well (Hegdé and Van Essen, 2000). The grating cell operator has not only shown great biological plausibility with respect to actual V2 texture processes but has also proven superior to Gabor filters in texture related tasks (Grigorescu et al., 2002). Its response is relatively weak to single bars but in contrast, it responds maximally to periodic patterns.

The approach used here (Petkov and Kruizinga, 1997) consists of two stages. In the first stage grating subunits generate on-center and off-center cells responding to periodicity much like retina cells. In the following stage, grating cell responses of a particular orientation and periodicity are added together, a process also known in neurons as spatial summation (Movshon et al., 1978).

A certain response *Gr* of a grating subunit at position *(x, y)*, with orientation θ and periodicity λ is given by Petkov and Kruizinga (1997):

(7) $$\text{Gr}{\left(\text{x},\text{ y}\right)}_{\theta ,\lambda }=\{\begin{array}{c} \text{1},\;if\;\forall \;n,\text{ M}{\left(x,\;y\right)}_{\theta ,\lambda ,\;n}\geq \;\rho \text{M}{\left(x,\;y\right)}_{\theta ,\lambda }\\ \text{0},\;if\;\exists \;n,\text{ M}{\left(x,\;y\right)}_{\theta ,\lambda ,\;n}<\;\rho \text{M}{\left(x,\;y\right)}_{\theta ,\lambda } \end{array}$$

where *n* ∈ *{-3 … 2}*, ρ is the threshold parameter between 0 and 1 (typically 0.9). The maximum activities of M at a given location *(x, y)* and for a particular selection of θ, λ*, n*, are calculated as followed (Petkov and Kruizinga, 1997):

(8) $$M{\left(x,\;y\right)}_{\theta ,\lambda ,\;n}=max\{\begin{array}{c} s{\left(x^{\prime },\;y^{\prime }\right)}_{\theta ,\lambda ,\varphi_{n}}\;|\\ n\frac{\lambda }{2}\text{cos}\theta \leq x^{\prime }-x<\left(n+1\right)\frac{\lambda }{2}\text{cos }\theta \\ n\frac{\lambda }{2}\text{sin}\theta \leq y^{\prime }-y<\left(n+1\right)\frac{\lambda }{2}\text{sin }\theta \end{array}$$

(9) $$\phi_{n}=\{\begin{array}{c} 0,\;n=\;-3,-1,\;1\\ \pi ,\;n\;=\;-2,\;0,\;2 \end{array}$$

and

(10) $$\text{M}{\left(x,\;y\right)}_{\theta ,\lambda ,n\;}=max\left(\text{M}{\left(x,\;y\right)}_{\theta ,\lambda ,\text{ n}}\right)$$

The responses at *M(x, y)*_θ, λ, *n*_ in Equation 9, are simple cell responses with symmetric receptive fields along a line segment 3λ. Essentially this means that there are three peak responses for each grating subunit at point *(x, y)* at a given orientation θ. This line segment is split in λ/2 intervals. The particular position of each interval defines the response of on-center and off-center cells. In other words, a grating cell subunit is maximally activated when on-center and off-center cells of the same orientation and spatial frequency are activated at point *(x, y)*. In Equation 10, ϕ_*n*_ is the phase offset and for values between 0 and π, it corresponds to symmetric center-on and center-off operations, respectively.

In the second part of V2 grating cell design, a response w of grating cell centered on *(x, y)* along orientation θ and periodicity λ, is the weighted summation of grating subunits with orientations θ and θ + π, as given below:

(11) $$\begin{array}{l} \;w{\left(x,y\right)}_{\lambda ,\theta }=\int exp\left(-\frac{{\left(x-x^{\prime }\right)}^{2}+{\left(y-y^{\prime }\right)}^{2}}{2{\left(\beta \sigma \right)}^{2}}\right)\\ \left(\text{Gr}{\left({\text{x}}^{\prime },{\text{y}}^{\prime }\right)}_{\theta ,\lambda }+\text{Gr}{\left(\text{x},\text{ y}\right)}_{\theta +\pi ,\lambda }\right)dx^{\prime }\;dy^{\prime },\;\theta \in \left[0,\pi \;\right) \end{array}$$

Parameter β is the summation area size with a typical value of 5. In our experiments the number of simple cells were empirically chosen at 3 and all other parameter values were set at default values according to Petkov and Kruizinga (1997).

### BIOPAD Structure

Light waves are being continuously perceived by our eyes and every generated electrical impulse passes via the lateral geniculate nucleus of our brain to arrive at the first neurons in the striate cortex (Hubel and Wiesel, 1967). Countless neurons organized in progressive layers then process this information through cascades of cerebral layer modules each intended for a specific operation. Broadly, visual areas in the human brain after visual area V2 follow the dorsal and ventral visual pathways, the “where” and “what” pathways (Schneider, 1969; Ungerleider and Mishkin, 1982). The two streams are layers along two distinct cerebral paths that localize and analyse meaningful information in constant neuronal communication.

BIOPAD's structure mimics the basic visual areas V1 and V2 in the primary visual cortex in a bottom-up fashion (Figure 2). Its operation relies on the early stages of biological visual cognition, without any external biases or influences. The design successively processes extracted biologically-inspired features reducing their dimensionality to an extent that they can be used with classifiers that determine original from fake access attempts. Furthermore, through successive biologically-motivated filtering BIOPAD's main strength lies in its ability to transform extracted features into higher dimensional vectors in a simple way that maximizes the separation between them. For example, an important difference between BIOPAD and HMAX is that the latter model's main focus is view-invariant representation of objects irrespective of their size, position, rotation and illumination. Conversely, BIOPAD's purpose is the detection of face spoofing attempts and to this end, invariance properties such as size and position could be valuable with future extensions. Even though invariance properties are generally meaningful in face recognition (Yokono and Poggio, 2004; Perlibakas, 2006; Rolls, 2012), in this particular scenario of face presentation attack detection they add unnecessary complexity or processing delays and are therefore not explored further. More specifically, BIOPAD's proposed structure is separated in the following layers (Figure 2):

**Input Layer:** The purpose of the input layer is to prepare image information by scaling down all input RGB images to a minimum of 300 pixels for the shortest edge in order to preserve the image's aspect ratio. This particular image size was chosen as a good compromise between speed/time and computational cost.

**Layer L1:** This layer plays the role of the lateral geniculate nucleus and separates visual stimuli in the appropriate double-opponency channels (bipolar cells) as given in section Area V1—Edge detection while scaling all pixel values to the same range between 0 and 1.

**Layer L2a:** Gabor filter operations perform edge detection according to parameterization values given in section Area V2—Texture features producing feature maps for each channel. It is important to note that after obtaining filtered outputs from all Gabor filters (in total 192) for each double-opponency channel, a maximum operator is applied so that a particular maximum response of L2a vectors *(x*_1_ …* x*_*m*_*)* in a neighborhood *j* is given by:

(12) $$r=arg{\text{ max}}_{\text{j}}({\text{x}}_{\text{j}})$$

**Figure 3:**
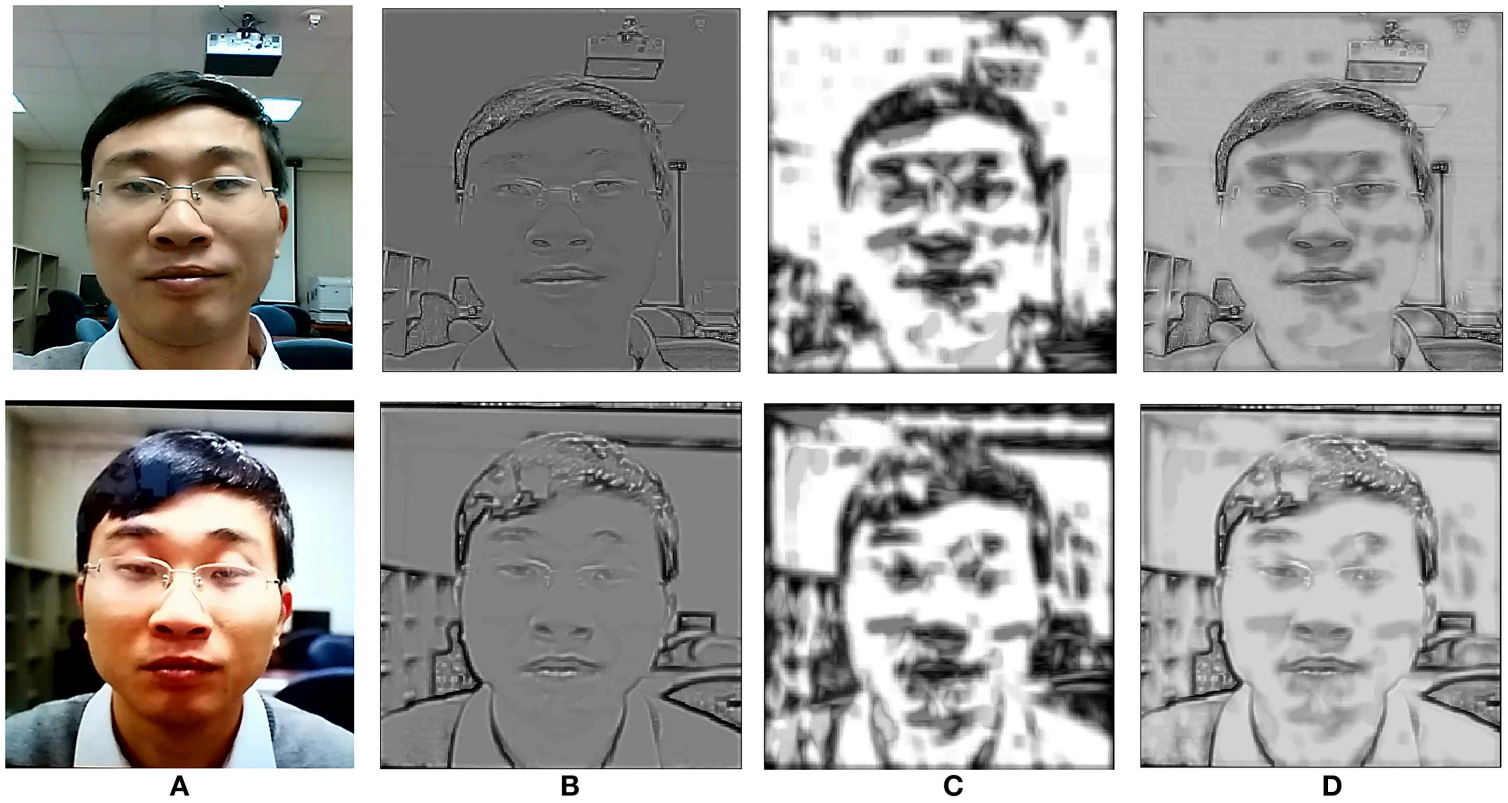
A genuine access attempt vs. a photo-print attack. Top row shows the progressive process of a genuine photo attempt. Bottom row shows the printed photo attack. Column **(A)** shows the input layer images. Column **(B)** the L2a layer as processed from edge detection Gabor filters, column **(C)** the L2b layer processed from texture grating cells and column **(D)** the combined layers L2a and L2b after spatial summation. The richness and depth of edge-texture information in the original image (top row) is apparent. All participants gave written informed consent for the publication of this manuscript.

The maximum operator is a well-known non-linear biological property exhibited by certain visual cells at low levels of visual cognition that assists in pooling visual inputs from previous layers (Riesenhuber and Poggio, 1999; Lampl et al., 2004) to greater receptive fields. This hierarchical process gradually projects meaningful visuospatial information to higher cortical layers in the mammalian brain (Figures 3a,b).

**Layer L2b:** In this layer grating cell operations are performed according to the settings given in section BIOPAD structure. Subsequently, grating outputs are spatially summed with outputs from L2a, in order to form a single L2 output for each of the three double-opponency channels. Spatial summation is another property of the visual cortex and like the maximum operator it is intended to linearly combine presynaptic inputs into outputs for higher layers (Movshon et al., 1978). Spatial summation is used in this layer in order to preserve the spatial integrity and sensitive texture information in faces (Figure 3c).

**Layer L3:** The three double-opponency channels after spatial summation (Figure 3d), contain both edge and texture features. The information of these channels along with the RG-BY spectral channels from L1 that contain the spectral differences of each image, are aggregated into spatial histograms with a window size of 20 units and bin size of 10. These values were empirically selected after experimentation as ideal for the particular layer dimensions. These spatial histograms have been used before in the context of face recognition but with lower level features at L1 (Zhang et al., 2005). Here, they are employed at an intermediate level of feature processing and with various types of biological-like features. It is further important to note here that since all these spatio-spectral channels carry different types of visual information, they are never mixed together.

**Layer L4:** In this layer all L3 information from the previous layer is simply concatenated and sorted in a multidimensional vector for either the training or testing phase, without any further processing. Vector dimensions vary according to the size of the dataset and choice of parameters within the model. For example, if from the previous L3 settings spatial histograms are performed over larger regions or if the input image layer of the image is set to smaller dimensions (for faster processing speeds), then the total number of vectors extracted will be smaller. Moreover, if the total number of images in the dataset changes, so does the vector dimension size, i.e., m_d_×n_p_, where *m* are the vectors extracted from previous layers with length *d* and *n* are the columns of vectors per image *p*.

**Layer L5:** Supervised classification takes place in this layer and any classifiers used can be trained with the extracted feature vector from L4. Training data are selected by following the 10-fold cross-validation technique. The supervised classifiers chosen for this work were a Support Vector Machine (SVM) with a linear kernel and k-Nearest Neighbor (KNN) with Euclidean distance.

BIOPAD's overall operation is further demonstrated with a pseudo-code approach below:

#### RGB Data Setup

Each PAD database consists of single **RGB** frame samples for a particular person's authentic video sequence and their presentation attacks. The PAD image database is then split in 70% training samples (*T*_*r*_) 30% samples for testing (*T*_*s*_) with cross-validation in *10-folds*.

***if*** *RGB case train* ***then***,

***for each*** *random T*_*r*_ *sample of each fold* ***do*,**

1. **Input:** Load a *m* × *n T*_*r*_ sample and scale to *300 pixels* for the shortest edge.
2. **Center-surround:** Convert **RGB** space to **O1, O2, O3** channel opponent space using Equations (2–4) thus obtain **opponency** frame *O*_*r*_ of the same dimensions.
  ***for*** *each opponency channel O1 (red –green differences), O2 (blue–yellow) and O3(lightness)* ***do***,
3. **Process V1:** Load *3x3, 5x5, 7x7, 9x9* ***Gabor filters*** ***(G***_***f***_***)*** parameterised with σ = *1, and* λ = *4, 5.6, 7.9, 11.31, 15.99, 22.61* in total *192* filters **then**.
  - *L1*_*Tr*_ = *O*_*r*_ · *G*_*f*_**,** where *L1*_*Tr*_ is a multidimensional array of *m* × *n* × *192* convolved versions of the *T*_*r*_ frame with V1-Gabor like filters.
  - Extract the maximum response using Equation (12) at every position along the dimension of convolutions to obtain a new matrix *L1*_*M*_
  - Normalize *L1*_*M*_ with zero mean and unit variance.
4. **Process V2**: Load grating filters (*G*_*r*_) using θ = *0–360*° in *45*° steps, λ = *5.42*, ρ = *0.9*, and β = *5*.
  - *L2*_*Tr*_ = *O*_*r*_ · *G*_*r*_, where *L2*_*Tr*_ is a multidimensional array of *m* × *n* × θ convolved versions of the *T*_*r*_ frame with V2 -grating filters.
  - Extract the maximum response using Equations (10–12) at every position along the dimension of convolutions to obtain a new matrix *L2*_*M*._
  - Normalize *L2*_*M*_ with zero mean and unit variance.
5. **Spatial summation** of *L1*_*M*_ *and L2*_*M*_ features yielding an array of the same size as the input.
6. **Spatial histograms** on summation output from step *5*, with a fixed window size of *20x20* L3 units and bin size of *10*, then concatenate histograms into a column of *5920* L4 vectors for each sample
7. Train classifier after all *T*_*r*_ have been processed through steps (1–6).***else if*** *RGB case test* ***then***,
  ***for each*** *random T*_*s*_ *sample of each fold* ***do*,**

repeat steps (1-6) as above and use *5920* column vectors of *T*_*s*_ to extract predictions from the trained classifier

#### RGB and NIR Data Setup

The FRAV database consists of **RGB** and **NIR** single samples for a particular person's authentic video sequence and their presentation attacks. The PAD image database is then split in 70% training samples (*T*_*r*_) 30% samples for testing (*T*_*s*_) with cross-validation in *10-folds*, maintaining RGB and NIR original sample ratios.

***if*** *RGB and NIR case train* ***then***,

***for each*** *random T*_*r*_ *sample of each fold*, ***do***

repeat steps (1-2) and (3-6). At L1 for each opponency channel O1 (red –green differences), O2 (blue – yellow), O3(lightness), NIR (near-infrared) extract *7100* L4 column vectors for each *T*_*r*_ sample during classifier training.

***else if*** *RGB and NIR case test* ***then***,

***for each*** *random T*_*s*_ *sample of each fold* ***do***,

repeat steps (1-2) and (3-6). At L1 for each opponency channel O1 (red –green differences), O2 (blue – yellow), O3(lightness), NIR (near-infrared) extract *7100* L4 column vectors of *T*_*s*_ for predictions obtained from the trained classifier.

## Experiments

It is important to note that in all experiments for both the genuine access and impostor attacks, only one photo per person was used from the entire video sequences. The databases employed in this work and their different spoofing attacks are explained in section Databases. Section Presentation attack results presents the obtained results in conventional biometric evaluation measures. The remaining part of this section is further divided into experiments in the visible and near-infrared spectrum. In this subsection, the different spectra are examined individually and subsequently, their cross-spectral fusion at feature, and score levels. Since our model currently does not perform any liveness detection method, successive video frames are not being considered. For the purpose of homogeneity and statistical accuracy between datasets, train and test data were divided with the cross-validation technique, bypassing the original train/test data split of some databases as has been explained in the previous section in more detail.

### Databases

The Facial Recognition and Artificial Vision (FRAV) group's “attack” database addresses several critical issues compared to other available face PAD databases. The number and type of attacks can vary significantly in each facial presentation attack database and by large, databases of the past never included a large sample of known threats. In addition to the sample of individuals examined being relatively small, little attention was paid in the multitude of human characteristics often occurring within human populations e.g., beards, glasses, eye color, haircuts etc. At the same time, sensor equipment is often limited and out-dated to contemporary technology products found in the market today. These shortcomings necessitated the creation of an up-to-date PAD facial database according to ISO/IEC and ICAO standards with a larger statistical sample, multi-sensor information and inclusion of all basic attacks. This database serves as a simulation stepping stone for experimentation ahead for any real-world situation and supplements the list of existing databases found publicly. The introduction of this new database from our group offers the following main characteristics and contributions:

- The largest PAD-ready facial database to date with 185 different individuals of both genders and various age groups.
- The largest collection of sensor data aimed at PAD algorithms. Four different types of sensors namely Intel's Realsense F200, FLIR ONE mobile phone thermal sensor, Sony A6000 ILCE-A6000 and a HIKVISION surveillance camera and therefore covering a range of spectral bands in the visible, near-infrared (at 860 nm) and infrared (800–1500 nm).
- Various spoofing attack scenarios examined, which include the following types of spoofing attacks:

1. Printed photo attacks with high resolution A4 paper.
2. Mask attacks from printed paper.
3. Mask attacks from printer paper with eye areas exposed an eye blinking effect.
4. Video attack with a tablet electronic device.
5. 3D Mask attack (to this day limited but will be expanded in the future)

**Figure 4:**
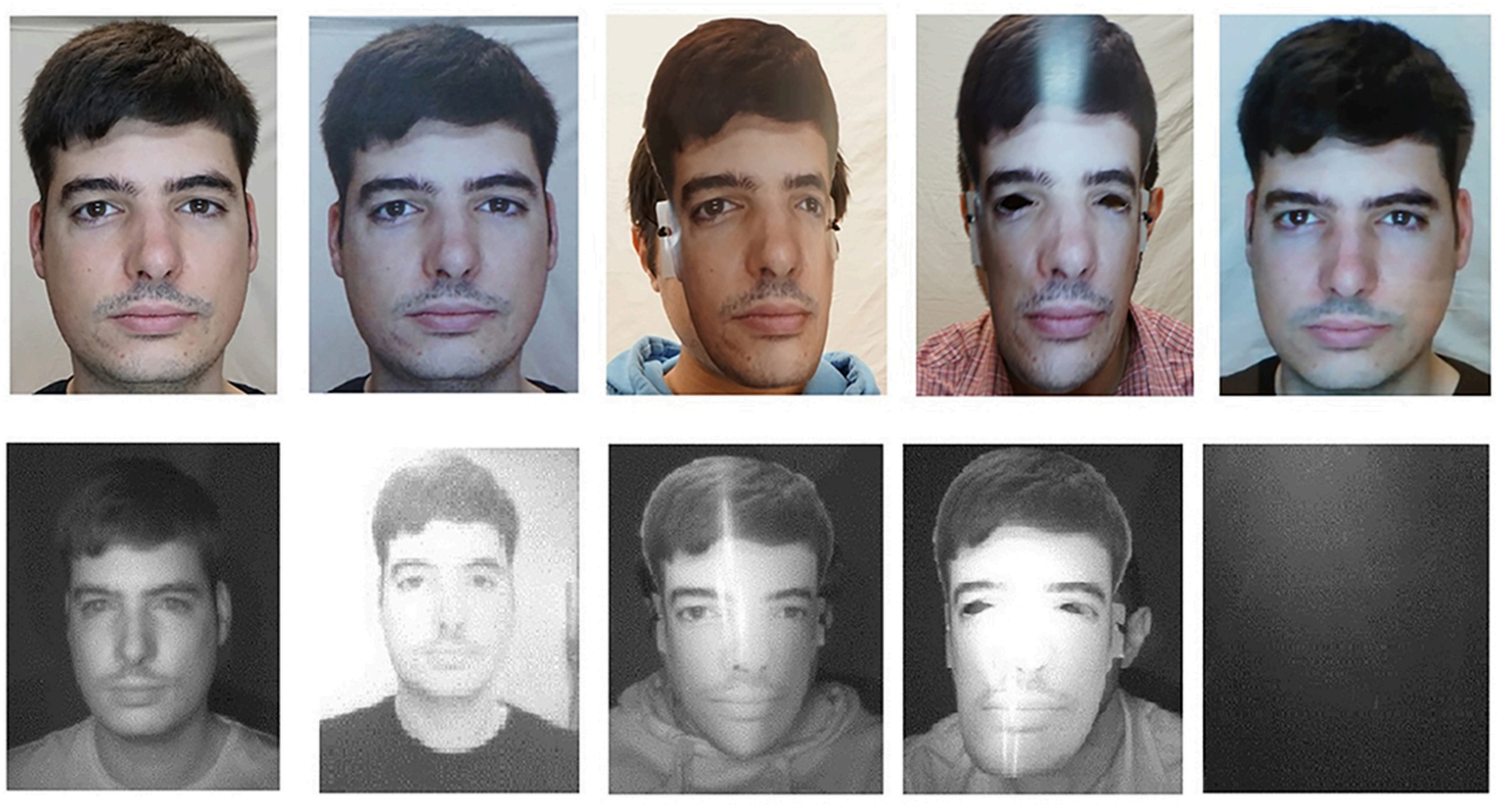
An example of a subject from the FRAV “attack” database. Top row left to right: Genuine access RGB photo, RGB Printed photo attack, RGB printed mask attack, RGB printed mask with eyes exposed attack, RGB tablet attack. Bottom row left to right: Genuine access NIR photo, NIR printed photo attack, NIR printed mask attack, NIR printed mask with eyes exposed attack, NIR tablet attack. All participants gave written informed consent for the publication of this manuscript.

Lastly, particular attention was paid at uniformly illuminating all faces using artificial lighting. Two T4 fluorescent tubes operating at 6,000 K−12 Watts each, evenly distributing multi-directional light to all subjects. Figure 4 illustrates all of the presentation attack types explored in the FRAV “attack” database for a given subject using RGB and NIR sensor information.

The CASIA Face Anti-Spoofing (Zhang et al., 2012) database is a database from the Chinese Academy of Sciences (CASIA) Center for Biometrics and Security Research (CASIA-CBSR). This database contains videos at 10 s of real-access and spoofing attacks of 50 different subjects, divided into train and test sets with no overlap. All samples were captured with three devices at different resolutions: (a) low resolution with an old 640 × 480 webcam, (b) normal resolution with a more up-to-date 640 × 480 webcam and c) high resolution with a 1920x 1080 Sony NEX-5 camera. Three different attacks were considered, (a) warped, spoofing attacks are performed with curved copper paper hardcopies of high-resolution digital photographs from genuine users, (b) cut, attacks are performed using hardcopies of high-resolution digital photographs from genuine users, with the eye areas cut out to simulate eye blinking, c) video, genuine user videos are replayed in front of the capturing device using a tablet.

The MSU Mobile Face Spoofing Database or MFSD (Wen et al., 2015) for face spoof attacks, consists of 280 video clips of photo and video attack attempts of 35 different users. This database was produced at the Michigan State University Pattern Recognition and Image Processing (PRIP) Lab, in East Lansing, US. The MSU database has the following properties, (a) mobile phones were used to acquire both genuine faces and spoofing attacks, (b) printed photos were generated as high-definition prints and their authors claim that these have much better quality than printed photos in other databases of this kind. Two types of cameras were used in this database, (a) built-in camera in MacBook Air at a resolution of 640 × 480, and (b) front-facing camera in the Google Nexus 5 Android phone at a resolution of 720 × 480. Spoofing attacks were generated using a Canon SLR camera, recording at 18.0 M pixel photographs and 1,080 p high-definition video clips and iPhone 5S back-facing camera, recording 1,080 p video clips.

### Presentation Attack Results

BIOPAD was evaluated with three different databases, FRAV-attack, CASIA, and MFSD. The main concern of our experiments was the detection success rate of spoofing attacks made by potential impostors. In simple terms, the system was required to effectively differentiate between fake and genuine access attempts. This was treated as a two-class classification problem. The applied biometric evaluation procedures are defined for the spoofing False Acceptance Rate (sFAR) and False Rejection Rate (FRR) as:

(13) $$sFAR=\frac{Impostor\;attacks\;seen\;as\;genuine}{Total\;number\;of\;attacks\;}$$

(14) $$FRR=\frac{Rejected\;genuine\;access\;attempts}{Total\;number\;of\;genuine\;access\;attempts}$$

Moreover, presentation attack detection is further presented according to SC37ISO/IEC JTC1 Biometrics (2014) with an additional measure, Average Classification Error Rate (ACER). The average of impostor attacks incorrectly classified as genuine attempts and normal presentation incorrectly classified as impostor attacks is given by:

(15) $$ACER=\;\frac{sFAR+FRR}{2}$$

Train and test data were partitioned using the *k*-fold cross validation technique. All scores were obtained using 10-folds and in order to further testify performance scores, and L4 feature vectors were essentially classified using two different schemas. A Support Vector Machine (SVM) classifier with two different kernels linear, Radial Basis Function (RBF) and a *k*-nearest neighbor (KNN) classifier of *n* = *2* nearest neighbors with Euclidean distance as a distance measure. In reality, the number of neighbors varies according to the dataset but for the two class problem here out of all *n* values examined, two produced the best average on all datasets as found through cross-validation. In the beginning, BIOPAD was examined only on the RGB images of all three databases and then on both RGB/Near-Infrared (NIR) images at feature-score levels for the FRAV attack database only since infrared data is unavailable for the other databases.

#### Visible Spectrum Experiments

Accuracy rates are defined as the number of images for each database correctly classified as genuine or fake, i.e., true positives and true negatives. The average classification accuracy scores and standard deviation values from all trials in Tables 1, 2, respectively, highlight the large differences between datasets and classifiers. From Table 1 it can be deduced that BIOPAD analyses presentation threats better than HMAX under all of the examined databases. Depending on the choice of training and testing data as provided by cross-validation, significant deviations in results may occur. This is largely due to the relatively small sample sizes in databases, especially in CASIA and MFSD, leading to significant statistical variance. This has an obvious effect on the KNN classifier which portrays an unstable and low performance with respect to SVM. The CASIA presentation attack database produced the worst overall results in terms of PAD.

**Table 1:** The average detection percentages (%) of 10 trials with cross-validation.

**Dataset**	**BIOPAD**			**HMAX**		
	**SVM linear**	**SVM RBF**	**KNN**	**SVM linear**	**SVM RBF**	**KNN**
CASIA	92.75	90.13	57.37	90.25	88.63	63.50
MFSD	97.08	86.04	82.08	90	87.08	70.42
FRAV	98.91	98.71	94.71	96.57	93.91	81.23

**Table 2:** The average standard deviation values (σ^2^) of 10 trials with cross-validation.

**Dataset**	**BIOPAD**			**HMAX**		
	**SVM linear**	**SVM RBF**	**KNN**	**SVM linear**	**SVM RBF**	**KNN**
CASIA	5.06	5.96	10.18	6.06	5.6	17.17
MFSD	3.82	3.68	9.97	7.84	9.86	11.23
FRAV	1.14	1.4	1.99	2.18	3.18	4.98

The highest performance has been achieved with the FRAV “attack” database closely followed by the performance achieved with the MFSD database. This is not entirely surprising since both datasets consist of good quality images and high resolution print attacks. The worst performance has been noticed when operating with CASIA photos. The total average performance from all datasets in the BIOPAD SVM linear case is at 96.24% while for HMAX at 92.27%. HMAX is not a dedicated PAD algorithm, nor has it been ever designed for such a purpose. Nevertheless, it can be seen from Table 1 that HMAX has performed remarkably well which beyond doubt proves the adaptability and capacity that bio-inspired computer vision models have.

In Table 2, standard deviation values further paint a picture of relationships between models and datasets. The highest performance was observed in BIOPAD with SVM using the FRAV database and the worst in HMAX KNN using CASIA. Between them there is a sizeable difference of 16% indicating the impact of choosing a particular scenario and classifier in PAD performance. It is further noticeable from this table that BIOPAD provides a more consistent set of results with SVM linear being the overall winner in performance. The detection accuracy rates in Table 1 provide an insight into the overall ability of the PAD model to detect spoofing attacks. From these results it is seen that the model can achieve a high detection rate at almost 99% with a consistent standard deviation value of 1.14 for the SVM linear kernel case in the FRAV database. Overall, the KNN classifier with the CASIA database has shown the worst performance. While conclusions from Tables 1, 2 are useful, biometric evaluation becomes more meaningful when measured in terms of sFAR and FRR which can effectively capture the nature of error.

In addition to HMAX and for a more complete comparison with BIOPAD, the selected databases were analyzed using Convolutional Neural Network. Multiple lines of research have been explored for CNN architectures in last two decades and a huge number of different methods are proposed in references (Canziani et al., 2016; Ramachandram and Taylor, 2017). In this part of the experiments, the objective is to compare the proposed bio-inspired method with a base line CNN model. The architecture selected was based on the well-known LeNet method (LeCun et al., 1998) with the improvements implemented in AlexNet (Krizhevsky et al., 2012). AlexNet has been tested for detecting presentation attacks using faces (Yang et al., 2014; Xu et al., 2016; Lucena et al., 2017). The architecture of the net is formed by eight layers, five convolutional and three fully-connected. All results provided in Table 3 are the average of 10 trials.

**Table 3:** AlexNet and BIOPAD average sFAR and FRR scores over 10 trials.

**Dataset**	**AlexNet**			**BIOPAD**		
	**sFAR**	**FRR**	**ACER**	**sFAR**	**FRR**	**ACER**
CASIA	2.857	13.9	8.37	2.77	14.58	8.67
FRAV	2.98	17.34	10.16	0.85	2.43	1.64
MFSD	9.64	39.07	24.34	3.44	5	4.22

Table 3 shows that error percentages are relatively small and comparable with another state-of-the-art algorithm like CNN that have been used in the past. The sFAR percentages for the CASIA and MFSD databases are comparable but there is a significant difference between the two databases in their FRR percentages. Naturally, this is also reflected onto the ACER percentages. The significant difference in FRR percentages indicates the difficulty of distinguishing attacks from genuine access attempts in the CASIA database. The error percentages for the best classifier choice (SVM linear) appear particularly improved for the FRAV attack database. In effect, this proves the importance of image quality in terms of both verification and presentation attack cases. Image quality is a consequence of various reasons and is also reflected in PAD results seen in Table 1. We further wanted to investigate the impact V1 and V2 edge and texture operations have on the overall performance of presentation attack detection. These tests were only performed for the SVM linear kernel case. It is worthwhile therefore to examine the separate and combined effect of V1 and V2 operations which can be seen in Table 4 below in terms of classification percentages. PAD scores rise when V1 and V2 feature vectors are combined together and standard deviation values across all trials indicate better performance. While these values are indicative in these early stages of experimentation, a separate study on optimum parameterization for each layer may yet reveal a more important relationship between edge and texture features in presentation attack detection.

**Table 4:** The average classification percentages (%) and standard deviation values of 10 trials with cross-validation for V1 and V2 operations.

**Dataset**	**μ**		**σ**^**2**^	
	**V1**	**V1 and V2**	**V1**	**V1 and V2**
CASIA	90	92.75	8.6	5.06
MFSD	95.63	97.08	6.25	3.82
FRAV	97.73	98.91	2.48	1.14

In order to better understand the intrinsic quality difference of the databases used in this work, various metrics were explored. There are numerous image quality metrics that have been developed over the years such as mean square error, maximum difference, normalized cross-correlation and peak signal-to-noise ratio amongst many others. Some of these metrics in fact have been successfully used as a separate PAD algorithm (Galbally et al., 2014). The majority of quality metrics requires the examined image to be subtracted from a reference image. This produces accurate error results only when the images are identical i.e., when the image content is identical. However, in practice face databases are a collection of images from various sensors at different angles. So in this particular case, sharpness metrics capable of measuring the content quality from a single image would be more suitable and useful. Likewise as before with quality metrics, there is a huge list of sharpness metrics being used in literature today, e.g., absolute central moment, image contrast and curvature, histogram entropy, steerable filters, energy gradients etc. An in-depth database quality analysis is beyond the scope of this work, and we have experimented with several sharpness metrics noting similar responses from all. Table 5, shows indicative sharpness results by using the spatial frequency quality (Eskicioglu and Fisher, 1995) metric which has been representatively chosen.

**Table 5:** Direct comparison of spatial frequency quality index values for three datasets and for each of their presentation attacks.

**Dataset**	**Printed photo**	**Printed mask**	**Printed Photo/Mask with Eye blinking**	**Smartphone**	**Tablet**	**Real users**	**μ**
CASIA	0.803	–	0.8957	–	1.0221	1.094	0.9538
MFSD	2.4191	–	–	2.7054	2.9603	2.754	2.7097
FRAV	1.8275	1.6544	1.5081	–	1.4906	1.831	1.6623

**Figure 5:**
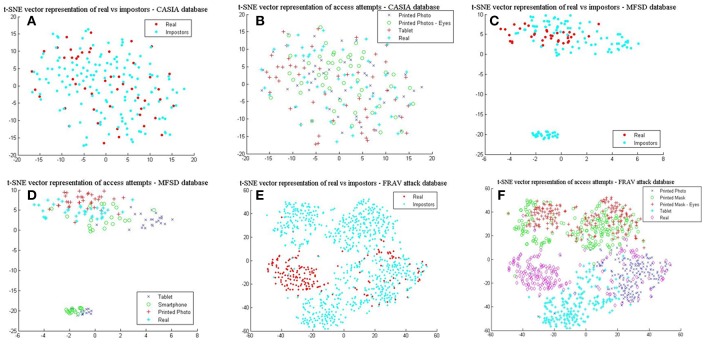
L4 vectors visualized with t-SNE for the three datasets. **(A)** real vs. impostors–CASIA database, **(B)** presentation attacks—CASIA database, **(C)** real vs. impostors –MFSD database, **(D)** presentation attacks—MFSD database **(E)** real vs. impostors—FRAV “attack” database, and **(F)** presentation attacks—FRAV “attack” database.

It is evident from the mean values (μ) in Table 5 that the CASIA dataset on average does not possess the high quality of spatial features seen in the MFSD and FRAV databases. Furthermore, the MFSD dataset has produced the best scores, however it should be highlighted that it does not have the same variety of presentation attacks found in the FRAV “attack” database nor the abundance of test subjects. The “Smartphone” and “Tablet” attacks are a similar type of electronic device attack and there is no provision of mask attack data. To further understand the importance of the aforementioned better, we employ the t-Distributed Stochastic Neighbor Embedding (t-SNE) (Van Der Maaten and Hinton, 2008) technique to visualize and compare presentation attacks in each dataset. L4 vectors as extracted from BIOPAD are used with t-SNE technique at “default” value settings, i.e., 30 dimensions for its principal component analysis part and 30 for the Gaussian kernel perplexity factor, and shown in Figure 5.

In Figures 5A,C,E, real access attempts vs. impostor attacks are visualized within the same space. These illustrations help understand how genuine users distance from their attacks. It can be easily observed in Figure 5A that for the CASIA dataset real access attempts are scattered across the same space as presentation attacks, making the classification process complex and difficult to achieve. This is also confirmed by its reduced detection rates. Different patterns are exhibited from results in Figure 5B, where real access attempts occupy a denser area within the impostor attack zone and finally in Figure 5C, in which real access attempts fall within a separate space. Looking at the presentation attack images in all datasets closely, it is not surprising to understand why these patterns occur. In Figure 5B, mainly due to the low image sharpness in CASIA (Table 5) and the nature of attack experiments, L4 vectors cover almost the same range of values and dimensional space. As the separation of presentation attacks and real access attempts improve in Figures 5D,F so do the results in Table 1. Finally, in Figure 5F, some real access attempts exhibit a noticeable overlap with their respective presentation attacks, particularly within the printed photo space, which is the main source of sFAR and FRR errors for the FRAV database. Arguably, the presentation attack that, in general, best matches genuine user information is the “printed photo” attack which can be efficiently faced in the NIR spectrum (section Near-infrared experiments and cross-spectral fusion).

**Figure 6:**
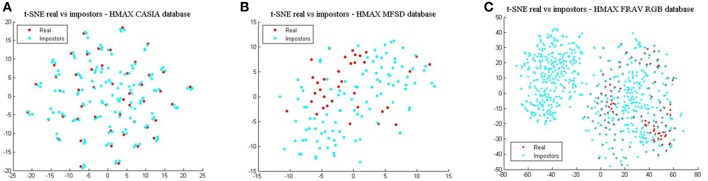
HMAX vectors visualized with t-SNE for the three datasets in terms of real access attempts vs. impostors. **(A)** t-SNE for the CASIA dataset, **(B)** t-SNE for the MFSD dataset, and **(C)** t-SNE for the FRAV “attack” dataset.

Finally, comparing BIOPAD L4 vectors with HMAX vectors using t-SNE (Figure 6), it can be noted that HMAX vectors do not display the same amount of consistency in distinct areas but rather vectors from all attacks appear merged and scattered across the same area. HMAX lack of bio-inspired features capable of processing texture and color information, leads to hardly distinguishable classes. In effect, this has a toll in presentation attack detection results (Table 1).

#### Near-Infrared Experiments and Cross-Spectral Fusion

BIOPAD experiments in the previous section have centered on the visible spectral bands and have shown great promise. Nonetheless, there were noticeable overlaps with certain presentation attacks and so we wanted to further expand BIOPAD's capacity to cope with these attacks and minimize the contribution of errors either directly from the subjects or their ambience. For this reason, our experiments in this section present a direct comparison between the performance for each spectral band, then their fusion at feature and score levels i.e., fusion between the visible and NIR band. At feature level, NIR is treated like an additional channel (Figure 2) and L4 vectors from all bands are equally processed in the model. Conversely, at score level visible—NIR bands are processed and classified separately. However, after classification, vectors for each subject are examined over all trials using the weighted sum score level fusion technique in order make a decision on whether the subject is genuine or not.

For this round of experiments, we only process the FRAV “attack” dataset since NIR data is unavailable in other datasets and to our knowledge the FRAV “attack” database is the only face presentation attack dataset in literature. Originally, the FRAV “attack” dataset consists of 185 different subjects and experiments in the previous section were conducted under this sample. In these experiments, available data for different subjects is changed to 157 individuals since there were failure-to-acquire instances during database acquisition. All other setup parameters remain unchanged as before.

**Figure 7:**
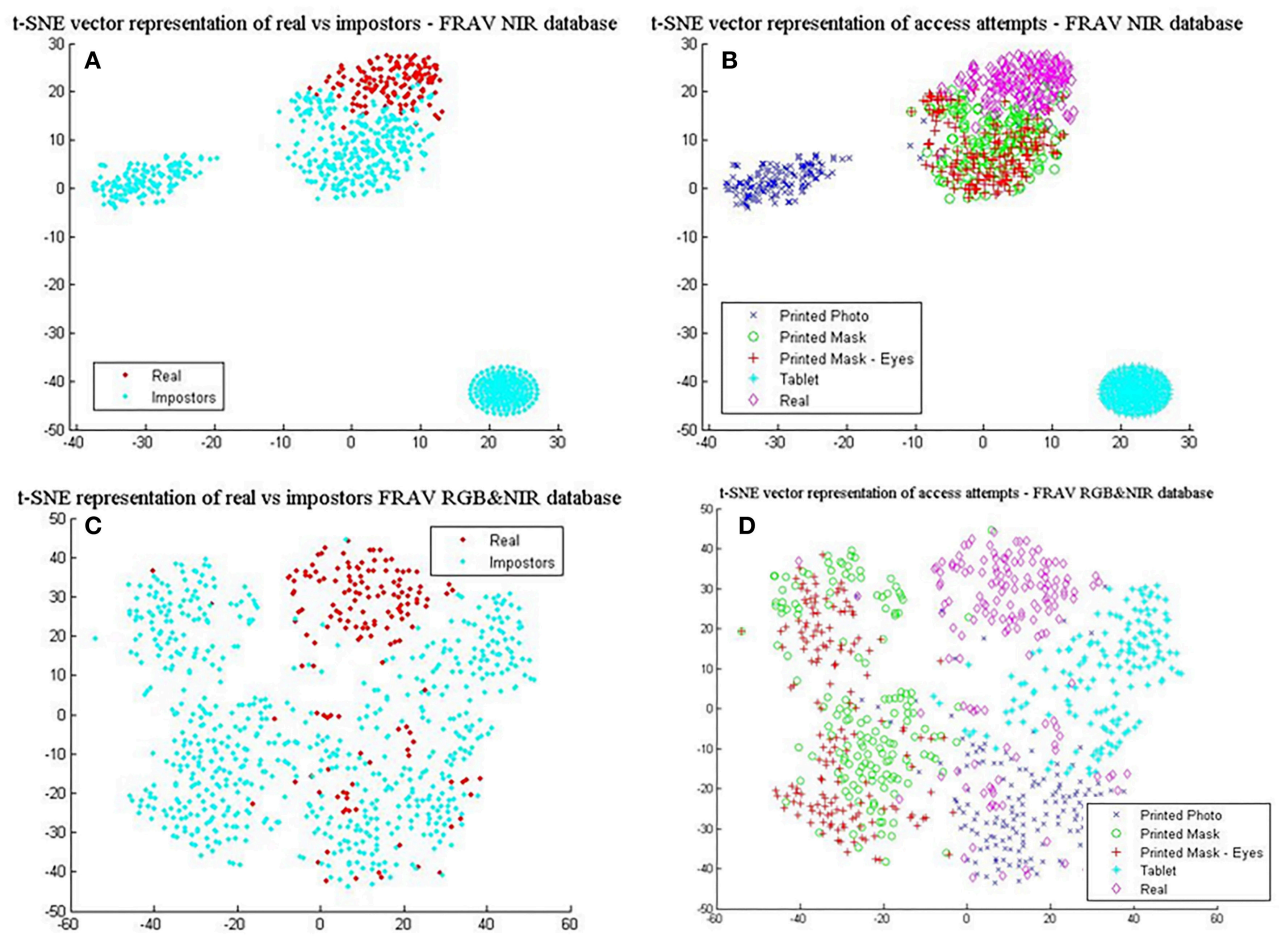
L4 vectors visualized with t-SNE for the FRAV “attack” database and its NIR information. **(A)** real vs. impostors—FRAV “attack” database with NIR information only, and **(B)** presentation attacks—FRAV “attack” database with NIR information only, **(C)** real vs. impostors—FRAV “attack” database with RGB&NIR information fused at feature level, and **(D)** presentation attacks—FRAV “attack” database with RGB&NIR information fused at feature level.

In Table 6, the best results with the least standard deviation values for BIOPAD across all classifiers were obtained by using NIR images. The drop in performance in the visible spectrum is nearly 1.5% for the SVM linear classifier case and this pattern trend is consistent with other classifier settings. NIR superiority in this type of presentation attack experiments can be further viewed from their t-SNE results in Figures 7A,B, where it is apparent that classes are well-separated. These representations can be directly compared with the visible spectrum case (Figures 5E,F) where there was a clear overlap between genuine and impostor attacks leading to errors being introduced in sFAR and FFR. The overlap between genuine access attempts and printed photo attacks does not exist in the NIR case and the “tablet” is completely neutralized since there isn't any useful attack information being projected at NIR. Fusing visual information between the visible and NIR at feature level, caused BIOPAD to lose slightly in detection rate performance with respect to NIR only by ~1.5%, also noticeable in standard deviation values. Moreover, when visualized at feature level and with the visible spectrum analyzed (Figures 7C,D), attack patterns appear slightly improved to Figures 5E,F but otherwise similar patterns are noticeable.

**Table 6:** BIOPAD detection rates and their standard deviation values over 10 trials.

**Dataset**	**SVM linear**	**SVM RBF**	**KNN**	**σ2-SVM linear**	**σ2-SVM RBF**	**σ2-KNN**
FRAV RGB	96.13	94.58	85.95	2.26	3.21	3.91
FRAV NIR	97.81	97.17	92.28	1.72	2.16	3.2
FRAV(RGB + NIR) Feature level	96.33	95.71	86.49	3.08	2.93	3.07
FRAV(RGB + NIR) Score level	96.97	95.87	89.11	1.99	2.68	3.55

**Figure 8:**
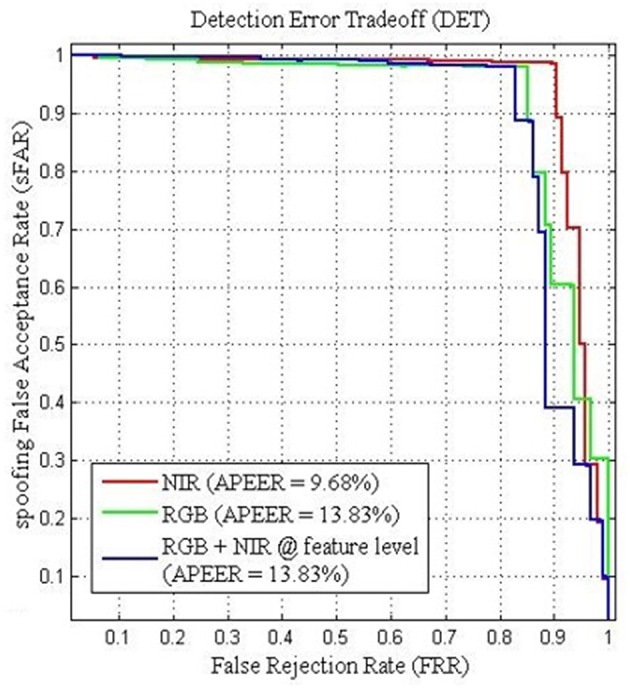
BIOPAD Detection Error Tradeoff curves of SVM linear classifier for the FRAV “attack” database in NIR(red), RGB + NIR at feature level (blue) and RGB (green). Attack Presentation Error Rate—APER.

Furthermore, the performance between the different visual information can be viewed from the Detection Error Tradeoff (DET) curve as shown in Figure 8. The DET curve for the FRAV “attack” illustrates the relationship within sFAR and FRR. Naturally, sFAR and FRR confirm the same behavior seen in the percentages, also presented in Table 6. As expected the best curve is obtained by BIOPAD with NIR followed by RGB + NIR (feature level) and RGB. Equal error rate or Attack Presentation Equal Error Rate (APEER) is a biometric security system indicator that determines the threshold values for sFAR and FRR. When these rates are equal, their common value is known as the “equal error rate.” This value specifies the proportion of false acceptances to false rejections. Low equal error rates mean higher accuracy. In Figure 8, the difference between APEERs in BIOPAD's case is 4.15% and undoubtedly shows that for the types of attacks present in the FRAV “attack” database, the best acquisition method for PAD is with the use of a NIR sensor.

## Conclusions

In this article we presented a novel presentation attack detection algorithm that relies on the extraction of edge and texture biologically-inspired features, by mimicking biological processes found in areas V1 and V2 of the human visual cortex. This model termed as “BIOPAD,” reproduced impressive presentation attack detection rates of up to 99% in certain cases by only utilizing one photo per person and for all attacks examined in the three datasets that were investigated. The main contributions of this research work were to (a) Present a novel biologically-inspired PAD algorithm which behaves comparably to other state-of-the-art algorithms. (b) Introduce a new PAD database called FRAV- “attack,” and (c) Introduce near-infrared band information for PAD experimentation at feature and score levels.

BIOPAD has been successful in surpassing other standard biological-like techniques such as HMAX and CNN which are considered state-of-the-art and benchmark models in biologically-inspired vision research. In addition, the creation, introduction and implementation of a new face presentation attack database by our group termed as “FRAV attack,” extended our investigation conclusions with high definition samples and diverse scenarios for the most commonly used spoofing attacks. The “FRAV attack” dataset which encompasses visual data that span from visible to infrared, is expected to set future standards for all new databases in face biometrics.

For the first time in literature, a biologically-inspired algorithm has been directly applied with near-infrared information, specifically for the purposes of face presentation attack detection. As observed from the experimental analysis in section Presentation attack results, BIOPAD features maximize the separation between attacks and as a consequence increase attack detection performance. The sFAR and FRR indicate that BIOPAD error performance falls within acceptable limits and it was further evident from our experiments that the nature of data were better separated in classification by a SVM linear classifier. However, future research in classification might reveal classification schema more effective in dealing with incoming data from multiple sensors.

Our results have also shown that near infrared sensor information is of extreme value and importance for presentation attack detection, significantly outperforming visible spectrum data. In our case, an increase in detection rate of almost 6% was observed between the near-infrared and visible scenarios. While the usefulness of near infrared information appears indisputable, we have proposed data fusion from multiple sensors to minimize errors from future elaborate attack methods that have not yet been investigated. To this end, data fusion at feature and score level indicate enhanced detection rates with respect to rates obtained from the visible spectrum.

Overall, results were promising and BIOPAD can serve as a foundation for further enhancements. Future work will include refinement of the biological-like operations to significantly increase performance and speed, optimization of presentation attack detection for video, and real time processes by incorporating biologically-inspired liveness detection algorithms, experimentation with multiple sensors, different types of novel and sophisticated presentation attacks, and experimentation in dynamic—real world situations.

## Ethics Statement

This study was carried out in accordance with the recommendations of the European Union, Spanish police, Spanish government, and University of Rey Juan Carlos with written informed consent from all subjects. All subjects gave written informed consent in accordance with the Declaration of Helsinki. The protocol was approved by the University of Rey Juan Carlos in Spain.

## Author Contributions

AT is the principal author, main contributor, and researcher of this work. CC helped in the following sections: original research, experiments, and text revision. BG helped during experiments. EC supervised this work and helped in the following sections: original research, during experiments, and text revision.

### Conflict of Interest Statement

The authors declare that the research was conducted in the absence of any commercial or financial relationships that could be construed as a potential conflict of interest.
